# Editorial: Biomechatronics: Harmonizing Mechatronic Systems With Human Beings

**DOI:** 10.3389/fnins.2018.00768

**Published:** 2018-10-25

**Authors:** Dingguo Zhang, Venketesh N. Dubey, Wenwei Yu, Kin Huat Low

**Affiliations:** ^1^School of Mechanical Engineering, Robotics Institute, Shanghai Jiao Tong University, Shanghai, China; ^2^Faculty of Science and Technology, Bournemouth University, Poole, United Kingdom; ^3^Center for Frontier Medical Engineering, Chiba University, Chiba, Japan; ^4^School of Mechanical and Aerospace Engineering, Nanyang Technological University, Singapore, Singapore

**Keywords:** biomechatronics, exoskeleton, prosthesis, rehabilitation robot, human-machine interface, brain-machine interface, electromyography, electroencephalography

There has been a growing body of research in the recent years on human-robot interactions, human-machine interfaces and intelligent devices that are centered around human application, however, these works by and large lacked in focus on how to harmonize the interactions between mechatronic systems and users in the loop. This is one of the key areas for evaluating the success of any mechatronic system implementation on human. The collection of papers in this volume is touching upon the frontiers of this research area as to how the efficacy of such biomechatronic systems could be evaluated and improved. There are a total of 19 papers looking into various aspects of human-machine interfaces (HMIs) using electromyography (EMG) and electroencephalography (EEG), tactile feedback, external devices such as exoskeletons and prosthetic devices for assistance and rehabilitation, novel techniques like machine learning and intelligent computation, and experimental evaluation or validation. The following paragraphs aim to give a glimpse of the contents presented in this eBook. Specifically, these are categorized under three distinct headings: (A) Novel exoskeletons for assistance and training, (B) Advanced human-machine interfaces in biomechatronics, and (C) Experimental outcomes and validation.

## A. Novel exoskeletons for assistance and training

There has been a surge of interests in implementing “soft robots” into every aspect of robotic assistance and training since it can provide compliance with inherent safety features to users. The paper by Koh et al. discussed a soft robotic elbow sleeve with full design features and functionality using elastomeric and fabric-based pneumatic actuators; what was more interesting was the intent based actuation control. The user intent was detected by surface electromyography (sEMG) on healthy subjects and the results showed that all participants were capable of achieving EMG control of the elbow sleeve. An extension of the above idea by the same group was explored by Yap et al. using soft robotic gloves with fabric-reinforced soft actuators, which targeted hand function assistance. They integrated elastic fabric with soft actuators to enhance the driven force for finger extension. A pilot test on stroke survivors showed improved grasping performance with the assistance of the soft glove. In a slightly different approach Oguntosin et al. have demonstrated functional properties of the exoskeleton actuated by soft modules. The exoskeleton has 3D printed parts with passive joints and utilizing the gravity compensation scheme to make it ultra-light. The exoskeleton was proposed for precise reaching control in neurorehabilitation for individuals with specific motor impairments. These papers highlighted the advantage of using soft modules for rehabilitation.

In an unusual way Huang et al. have studied a cable based robot for assessment of motor control during 3D movements tracking with position varying gravity compensation. They have shown that there was a significant difference in control strategies with and without gravity compensation which might assist subjects in performing optimal movements during rehabilitation. Fang et al. have realized the fact that people swing their arms synchronously with leg movement in the normal gait. So they developed a rotational orthosis for walking with arm swing for gait training to provide coordinated interlimb performance. This could potentially improve gait rehabilitation outcomes of patients undergoing training; the device was tested for functional assessment with normal subjects and was reported to provide a stronger feeling of walking with arm swing than without.

## B. Advanced human-machine interfaces in biomechatronics

Advanced human-machine interfaces (HMIs) are the key to harmonizing mechatronic systems with human beings in the loop; many developed systems simply fail because they lack of good interfaces for interpreting user motion intention/states and providing tactile feedback in the systems. Liu et al. have developed a model for continuous and simultaneous decoding of multi-joint dynamic arm movements based on multi-channel surface EMG signals. This was used to myoelectrically control exoskeletons for upper-limb rehabilitation which showed the potential for effortless control of impaired arms. On a similar approach Zhang et al. have used sEMG signals for simultaneous and continuous estimation of shoulder and elbow kinematics using principal/independent component analysis and artificial neural network for learning electromechanical association. This was done to achieve natural and intuitive human-machine interaction particularly for applications in exoskeletons, prostheses and other arm rehabilitation techniques. In an extension to the above approach Wu et al. developed grip force and three-dimensional push-pull force estimation based on sEMG and generalized regression neural network. This work was proposed to meet the requirements of force control of the intelligent prosthetic hand which employed EMG and grip force sensors to measure the output force of the human hand. Since all the above reported work employed EMG sensors, there must be a better way of acquiring stable signals. Jiang et al. have proposed a Polypyrrole-coated nonwoven fabric sheet for designing EMG sensors. The design can be customized for shape and size and can be sewn onto elastic band for close contact with skin for practical control of prosthetic hand. The sensors provided comparable results with conventional Ag/AgCl electrodes typically used in EMG sensors.

In a slight depart from the EMG based HMIs using muscle signals, EEG based HMIs employ brain signals, which are well-known as brain-machine interfaces (BMIs). Some previous non-invasive BMIs adopted Fourier based methods for time-frequency decomposition for feature extraction of EEG. However, this technique was challenging for multi-channel EEG signals. To improve performance of multi-channel EEG classification, Wang and Veluvolu developed evolutionary algorithms for optimizing the features to reduce the overall dimensionality and preserve the class-related information. Their results show that the combination of Fourier based method with covariance matrix adaptation evolution strategy has the best overall performance. Compared with non-invasive BMIs based on scalp EEG, invasive BMIs use high-quality brain signals, which are crucial for advanced control of dexterous robotic arms or prosthetic hands. Ma et al. have conducted a trial with monkeys to decode lower limb muscle activity and kinematics from cortical neural spike recordings during stand/squat movements. They provided detailed analyses of recorded data and new insights into invasive BMIs that could be used for controlling complex biomechatronic systems.

To explore the mechanism of tactile sensation under transcutaneous electrical nerve stimulation, a 3D computational model was developed for estimating tactile nerve fiber excitability (Zhu et al.). The simulation showed comparable results with psychophysical experimental data with healthy subjects. This study has potential for providing tactile feedback for prosthesis and realizing bi-directional neural-machine interfaces in future. In order to improve challenge/skill ratio in a multi-modal interface for human-robot interaction, Rodriguez-Guerrero et al. have investigated simultaneous adaptation of game difficulty and haptic assistance through psychophysiological signals (heart rate, skin conductance level, and skin conductance response frequency) and performance feedback. They came up with a new matric (FlowIndex) to numerically quantify and visualize the challenge/skill relation which improved the balance between augmented performance and user satisfaction. Different from the traditional diagnosis based on the patient's self-description, Chu et al. have developed a method of pattern recognition to classify the pain intensity based on multiple physiological signals (blood volume pulse, electrocardiogram, and skin conductance level). This has implications for the patients with major cognitive and communicative impairments who are not able to describe their level of pain. The experimental results on healthy subjects showed that the method can provide objective and quantitative evaluation of pain intensity.

## C. Experimental outcomes and validation

The level of harmonization between mechatronic systems and human beings could not be assessed without validating these devices under experiments with human subjects. There are several studies that focused on experimental trials to prove the efficacy of their devices. For example, Crea et al. have reported Phase II of clinical validation of a powered exoskeleton for the treatment of elbow spasticity. The rehabilitation outcomes from 17 post-stroke patients with robotic elbow exoskeleton showed that intensive early treatment prevented the occurrence of spasticity at a later stage. Buzzi et al. proposed a non-disruptive method to study the arm endpoint stiffness in a teleoperated robotic system. Based on a musculoskeletal model, they could use arm kinematics and muscular activation (sEMG) to estimate the endpoint stiffness. Here, they just focused on experimental study to investigate how different master devices and tasks influence the regulation of arm endpoint stiffness and its relation with task performance, hand speed and acceleration. This experimental study may further help to understand which characteristics and parameters play a fundamental role in compliant human-robot interactions. In order to generate sufficient and accurate motion data for positive rehabilitation using exoskeletons, Liu et al. conducted experimental study based on their custom-made Neo-Arm exoskeleton. At each joint of Neo-Arm, an angular displacement sensor was equipped through the axis of the rotation, by which the angular information could be captured. Their methods have been compared with the conventional method i.e., a camera-based (Vicon) system. The experiments were conducted on 8 healthy subjects performing a series of movements, including five actions and five hovering postures, and the results showed that the satisfactory data could be achieved with suitable precision for upper limb motion tasks without the need for platform based systems. Since motor learning is a critical component of the rehabilitation process, Jarrett and McDaid conducted a simulated pilot study with healthy subjects to evaluate motor learning in case of physical impairment. They developed a technique where simulated impairment is applied to constrain the motion, such that motor learning could be studied independently of physical impairment. Their tool could be used to get insight into underlying causes of motor learning deficits. This could be used to provide subject–specific therapy. Jasni et al. have analyzed the interaction among the voluntary and mechanical joints and segments in users wearing lower-limb prosthesis/orthosis using cyclograms. The cyclogram model was developed using data from 20 healthy able-bodied subjects and 25 prosthesis and orthosis users (10 transtibial amputees, 5 transfemoral amputees, and 10 orthosis users).This cyclogram model enables quantitative judgment of the effect of changing a particular parameter in the prosthetic leg gait. This experimental study is particularly useful for understanding coordinated movement of several joints and limb segments wearing external devices.

So looking at the collection of papers in this research topic we are happy to endorse that this eBook provides a comprehensive appraisal of the latest developments in the field of biomechatronics including exoskeletons, prosthesis, and advanced human-machine interfaces and followed by experimental validation of some of the developed techniques. We hope that these papers will provide readers new insights into the aspects of harmonizing mechatronic systems with human beings.

## Author contributions

DZ was invited to prepare this research topic. He invited VD, WY, and KL to be co-editors in it. They all contributed to soliciting papers, inviting guest-authors, revising their manuscripts, and handling revisions with invited reviewers. Specifically, DZ edited 12 papers, VD edited 2 papers, WY edited 4 papers, and KL edited 1 paper. VD drafted the editorial.

### Conflict of interest statement

The authors declare that the research was conducted in the absence of any commercial or financial relationships that could be construed as a potential conflict of interest.

